# Epidemiology of Cervical Cancer in the Caribbean

**DOI:** 10.7759/cureus.48198

**Published:** 2023-11-03

**Authors:** Jamie Scott-Williams, Amalia Hosein, Patrick Akpaka, Chalapathi Rao Adidam Venkata

**Affiliations:** 1 Biomedical Engineering, The University of Trinidad and Tobago, Port of Spain, TTO; 2 Pathology/Microbiology, The University of the West Indies, St. Augustine, TTO; 3 Pathology and Laboratory Medicine, The University of the West Indies, St. Augustine, TTO

**Keywords:** review, mortality, incidence rate, public health, caribbean, cervical cancer

## Abstract

Cervical cancer (CvC) is considered a preventable disease; however, in the Caribbean, it is still one of the fourth most common causes of death in women. Efforts to overcome obstacles to the treatment and control of this preventable disease are being made by several countries within the Caribbean. However, no health issue can be readily managed without first acquiring an understanding of the dynamics relating to its severity of impact reaching the target population, its clinical pathology, and the availability of treatment and/or preventative measures to control or halt its progression. To assess the status of CvC in the Caribbean, a review of the literature was conducted using PubMed. The Caribbean was defined in the review as comprising nations and islands whose coastlines are touched by the Caribbean Sea. This led to an assessment of the available literature on CvC for 33 Caribbean territories. The review showed a lack of published information on CvC and highlights the need for greater research. This also serves as a template for subsequent investigations.

## Introduction and background

In 2020, CvC was listed as the fourth most common malignancy in women globally, as well as being widespread and fatal [1,2]. The relationship between CvC and the human papilloma virus (HPV) virus is widely revered as an important role in the prevalence and incidence. The advancements in treatment and prevention (e.g., vaccination) and management methods have improved through the years, and it is hoped that this ranking will decrease [3]. However, data for many Caribbean territories are lacking on incidence, mortality, patient demographics, clinicopathology, behavioral risks, genetics, and control and treatment [4].

CvC is considered preventable, and as such, the necessity of an early diagnosis is considered the main course of management [5]. This has led to greater focus being applied to the identification of abnormal cervical cells (abnormal pap-smear results) during the process of screening. Globally, in the early 2000s, the incidence of CvC had noticeably decreased as improved methods of screening were being introduced and implemented. The dissemination of information regarding these methods and their perceived benefits became a priority, and the relevance of cervical cytology as an important tool of preventative care became more known [3]. To achieve this, at the GLOBOCAN Conference by the World Health Organization in 2018, health experts initiated talks to align HPV testing as a co-screening method to the cytological Papanicolaou smear screening procedure as a response to the growing mortality rates due to CvC. This resulted in a global call for the ‘elimination' of CvC [2], which was subsequently reaffirmed at the GLOBOCAN 2020 conference.

However, it was observed that, among lower-income countries, there was a disparity in the availability and accessibility of screening and prevention programs [4]. This disproportion existed between the provision of information and the actual utilization of screening methods at varying socioeconomic levels [4]. Additionally, there were noticeable hindrances brought on by cultural attitudes regarding the screening procedure itself, such as the lack of spousal support and anxiety due to participation in the screening procedure [5].

To this end, a combination approach of primary and secondary treatment was considered most effective. This involved informed consent for the administration of the prophylactic HPV vaccine and the introduction of screening using HPV assays and Pap smears, as part of a female’s routine check-up, respectively [2]. The data showed that, at least 84% of deaths due to cancer, were caused by CvC in lower-income nations; as such, greater efficiency in treatment and prevention is needed in these regions by providing equal access to uniform methods of vaccination and screening [2,6].

There are three main HPV vaccines commercially available targeting HPV genotypes 16 and 18 [7-9], which have been identified as the main etiology for CvC. These are the bivalent (genotypes 16 and 18 only), quadrivalent (genotypes 6, 11, 16, and 18), and nonvalent (genotypes 6, 11, 16, 18, 31, 33, 45, 52, and 58) [8,10]. However, there are over 120 identified HPV types, 14 of which are considered high risk [9]. Therefore, further investigations into these HPV types may lead to causes directly or indirectly rooted in other forms of malignancy within the human reproductive system. By achieving a better understanding of these agents, a more direct investment can be made by intrinsically targeting the root of the actual problem. This suggests that an overall epidemiologic study of CvC and its main contributor (HPV) was required to build a comprehensive model for its management. Understanding the disease burden metrics, such as the incidence and prevalence of CvC in the Caribbean, survival and mortality rates, and consideration for the years of life lost, as well as the disability-adjusted life years of those affected, served as the foundation for this. This literature review's objective was to get a clear understanding of the presence and absence of these factors while taking into account any potential consequences, such as the cost of fertility preservation and affordability of treatment and vaccinations, in addition to the level of adherence in the Caribbean.

## Review

Methodology

**Figure 1:**
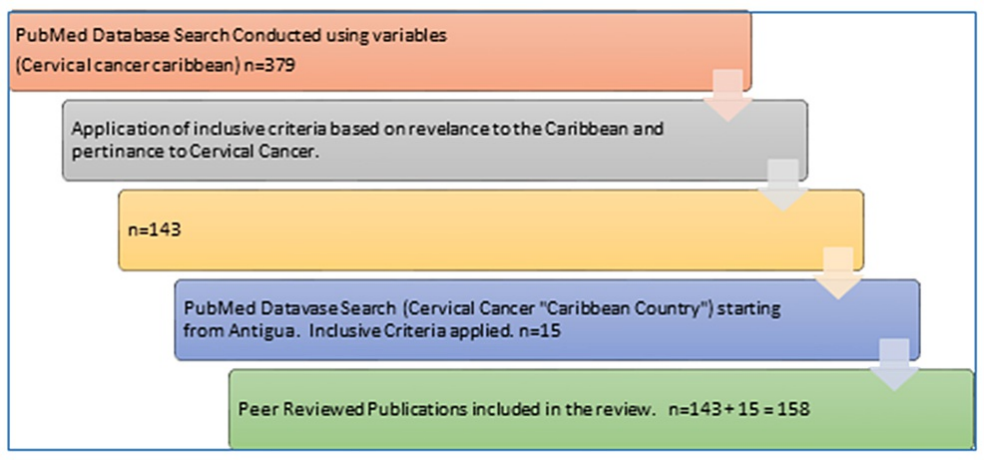
The Progression of the PubMed Search Conducted to Procure the Publications for the Review.

The PubMed search engine was selected to conduct a literature review on the information that is available from peer-reviewed publications on the topic of CvC, its contributing factors, development, progression, and prevention. This literature review was conducted within the last 63 years (1958 to October 2022) resulting from the key search words “cervical cancer in/and the Caribbean” on the PubMed database, which resulted in 379 records. Abstracts and full texts were reviewed for the inclusion eligibility of (1) all publications must discuss the Caribbean in general, or a specific Caribbean territory (composed of all territories listed in Table 1 below), and (2) the publications must be pertaining to an aspect of CvC, as indicated above. All studies found that were focused on CvC within the defined Caribbean population were included in Table 1, indicating the section of information covered. In addition, searches of each Caribbean territory by name and 'cervical cancer' as its search subject were conducted and resulted in 15 additional publications that fit the inclusion criteria. Finally, a total of 158 articles that met the inclusion criteria were included in this review (Figure 1).

**Table 1:** Information Coverage of the Publications Reviewed. x indicates the presence of the disease burden measure under consideration

Caribbean Territories	Location	Number of Articles	Incidence	Mortality	Patient Demographics	Clinico- pathology	Behavioral Risks	Genetics	Control	Treatment	Total Measures Covered
Anguilla	Northern Caribbean	1						x			1
Antigua and Barbuda	Northern Caribbean	0									0
Aruba (Dutch Caribbean)	Southern Caribbean	0									0
Bahamas	Northern Caribbean	9	x	x	x	x	x	x		x	7
Barbados	Eastern Caribbean	7	x	x	x		x	x	x	x	6
Belize	Central America	3	x	x				x	x	x	5
Bermuda	Northern Caribbean	0									0
Cayman Islands	Western Caribbean	0									0
Cuba	Western Caribbean	6	x	x	x		x	x			5
Curaçao (Dutch Caribbean)	Southern Caribbean	4			x	x	x	x	x		5
Dominica	Eastern Caribbean	0									0
Dominican Republic	Northern Caribbean	4	x	x	x	x	x		x		6
French Guiana (French West Indies)	South America	4	x	x	x	x	x	x			6
Grenada	Eastern Caribbean	1	x	x			x				3
Guadeloupe	Eastern Caribbean	4	x	x				x			3
Guyana	South America	4	x	x	x	x		x	x	x	7
Haiti	Northern Caribbean	10	x	x	x	x	x	x	x	x	7
Jamaica	Northern Caribbean	13	x	x	x	x	x	x			6
Martinique (French West Indies)	Eastern Caribbean	7	x	x	x		x	x			5
Montserrat	Eastern Caribbean	0									0
Navassa Island	Western Caribbean	0									0
Netherlands Antilles	Southern Caribbean	30	x	x	x		x	x	x	x	7
Puerto Rico	Northern Caribbean	29	x	x	x		x	x	x		6
St. Barthelemy	Northern Caribbean	0									0
St Kitts and Nevis	Eastern Caribbean	1			x		x	x			3
Sint Maarten (Dutch Caribbean)	Northern Caribbean	0									0
St Lucia	Eastern Caribbean	0									0
St. Martin	Northern Caribbean	0									0
St Vincent and the Grenadines	Eastern Caribbean	1			x		x	x			3
Suriname	South America	8	x	x	x			x	x		5
Trinidad and Tobago	Southern Caribbean	11	x	x	x		x	x	x	x	7
Turks and Caicos Islands	Northern Caribbean	0									0
United States Virgin Islands	Northern Caribbean	1							ü		1
Total Number of Articles Reviewed		158	144	144	148	48	137	152	61		

Results

As shown in Table 1, eight measures of disease burden were identified specific to CvC [10], including incidence, mortality, patient demographics, clinicopathology, behavioral risks, genetics, and control and treatment, and the presence of each measure type was indicated. For references of relevant findings and observations that were noted throughout the review, the attached citations can be found under the corresponding table. From Table 1, Puerto Rico and the Netherlands had the highest number of accessible publications with 29 and 30 articles, respectively. Jamaica, Trinidad and Tobago, and Haiti were less than half that value with 13, 11, and 10 publications, respectively. The Bahamas, Suriname, Martinique, Barbados, and Cuba followed with just over five, with the other regions trailing behind with four or fewer relevant documents. Six disease burden measures were covered by Barbados, the Dominican Republic, the French West Indies, Jamaica, and Puerto Rico, while five disease burden measures were covered by Belize, Cuba, the Dutch Caribbean, and Suriname. Three or fewer were covered by other areas. The Bahamas, Guyana, Haiti, the Netherlands, Trinidad and Tobago, Barbados, the Dominican Republic, the French West Indies, Jamaica, Puerto Rico, Belize, Cuba, the Dutch Caribbean, and Suriname covered seven disease burden measures despite no one territory covering all eight indicators.

Incidence

CvC incidence statistics for the Caribbean were patchy and inconsistent. Although Table 2 listed new CvC cases from 1958 to 2018, much of the data for the years in between was not accessible. The Bahamas underwent a continuous rise in newly diagnosed cases from 19 cases in 1993 to 28 cases in 1994 and 40 cases in 1995. There was no further data until 2018 when the number of cases dropped to 29 cases, which was around the same number as that in 1994. From an average of 24 instances per year during the period 1958-1964 to 38 cases in 2018, Barbados had a gradual rise in cases. Although statistics for the intervening years were not provided to allow for a more thorough examination of its development throughout that time, it represents a noteworthy rise of 14 instances over a span of 50 years. The annual detection of new instances of CvC appears to be actively monitored in the Netherlands. It was noted from Table 2 that the prevalence of CvC in the Netherlands had generally remained high, averaging 731 newly diagnosed cases each year from 1989 to 1998. The number of instances climbed the next year by 10 cases after a notable decrease of seven cases in 1994. Then, there was another drop of six instances, followed by another rise of 10 cases. Martinique experienced a significant decline in cases from an average of 45 cases per year (1981-2000) to an average of 25 cases per year (2008-2012) and then increased again to 32 in 2018. This showed that whatever measures were implemented during that period was perhaps not sustainable. From the period (1980-2000) to 2018, Suriname had an almost doubling of instances, from an average of 45 cases per year to 85 cases. Similarly, Trinidad and Tobago endured an increase from an average of 119 cases per year during the period (1995-2009) to 140 cases in 2018 and Puerto Rico increased from an average of 241 cases per year during the 2007-2012 period to 262 in 2018. Puerto Rico's growth was particularly concerning because there were already over 200 new cases reported annually, which is a considerably high level.

**Table 2:** The Cervical Cancer Incidence Data (CASES) Provided by the Peer-Reviewed Publications. Bahamas [11-13], Barbados [14,13], Belize [2], Cuba [2,5,13,15], Dominican Republic [2,5,13], French Guiana [2,5,13], Grenada [16], Guadeloupe [2,5,13], Guyana [2,5,13], Haiti [17], Jamaica [2,5,13,18], Martinique [19,20], Netherlands Antilles [21,22], Puerto Rico [2,5,13], Suriname [23,24], Trinidad and Tobago [25,26]

	Number of Newly Diagnosed Cervical Cancer Cases Per Year																	
Caribbean Territories	1958-1964	1980-2000	1981-2000	1988-2002	1989	1990	1991	1992	1993	1994	1995	1995-2009	1996	1997	1998	2007-2011	2008-2012	2018
Bahamas				58					19	28	40							29
Barbados	119																	38
Belize																		46
Cuba																		1231
Dominican Republic																		981
French Guiana (French West Indies)																		29
Grenada																	104	29
Guadeloupe																		39
Guyana																		124
Haiti																		835
Jamaica																		486
Martinique (French West Indies)			890														129	32
Netherlands Antilles					719	761	737	752	722	715	725		719	729	733			
Puerto Rico																1215	1203	262
Suriname		892																85
Trinidad and Tobago												1795						140

Age-standardized incidence rates (ASIR) were reported in 18 publications for 16 Caribbean territories (Table 3). The ASIR observed by the Netherlands showed that there was a significant reduction from 15.7 per 100,000 women in 1989 to 13.6 per 100,000 women in 1998. At 148 per 100,000 women, Cuba had the highest incidence rate in 1990. The next year, it rose dramatically to 183.6 per 100,000 women, and it continued to grow until 2000 when it reached 225.9 per 100,000 women. From there, it had a little decline to 224.2 per 100,000 women in 2006. Trinidad and Tobago was ranked 18th in the region by Andall-Brereton, for having an ASIR of 27.1 per 100,000 women in 2002. No information was provided thereafter for the years preceding 2011 when this rate was indicated to be the same. Then, in 2018, the ASIR was determined to be 15.2 per 100,000 women, which is a considerable decrease. Table 2 shows an average of 119 cases annually for the years 1995 to 2009, which corresponds to the rate of 27.1 cases per 100,000 women previously mentioned. It can therefore be concluded that the population size had significantly increased by 2018 to allow for a larger number of newly diagnosed cases (140), which is indicative of a much lower rate of 15.2 cases per 100,000 women. Comparatively, it is concerning that the ASIR for Suriname increased by 4.4 per 100,000 women during the subsequent eight years to a whopping 26.8 per 100,000 women in 2018, following a reasonably stable period from 1981 to 2010 where its ASIR was 22.4 per 100,000 women. In a similar vein, it was found that Guadeloupe's ASIR had grown significantly from 3.3 per 100,000 women in 2018 to 7.9 per 100,000 women in 2020 [11,27]. Nevertheless, compared to the other nations, Suriname and Guadeloupe both exhibited comparatively low ASIR levels. On the other hand, Jamaica's rate ranged throughout the past 30 years between 0.4 and 1.0, with the lower rates of 0.6 being more recent. This suggests that, even though Jamaica's incidence rate is low, CvC prevention and control efforts are not very successful.

**Table 3:** The Cervical Cancer Incidence Data (Age Standardized Rates) Provided by the Peer-Reviewed Publications. Bahamas [11-13], Barbados [14,13], Belize [2], Cuba [2,5,13,15], Dominican Republic [2,5,13], French Guiana [2,5,13], Grenada [16], Guadeloupe [2,5,13], Guyana [2,5,13], Haiti [17], Jamaica [2,5,13,18], Martinique [19,20], Netherlands Antilles [21,22], Puerto Rico [2,5,13], Suriname [23,24], Trinidad and Tobago [25,26]

Year Distribution of Cervical Cancer Incidence ( Age Standardized Rates/Per 100,000 Women)																									
Caribbean Territories	1981-2002	1988-2002	1989	1990	1995	1995-2009	1998	2000	2000-2009	2001	2001-2021	2002	2003	2004	2005	2006	2007	2007-2011	2008	2008-2012	2009	2010	2010-2011	2011	2018
Bahamas		60										16.7													10.9
Barbados												24.9													15.5
Belize		54.9										52.4													28
Cuba				148	183.6			225.9				20.2				224.2									14.6
Dominican Republic												30.8													17.1
French Guiana (French West Indies)																				20.7					20.8
Grenada									24.4																
Guadeloupe																									3.3
Guyana												47.3													32.7
Haiti												87.3		94											17.1
Jamaica		17.4																							28.4
Martinique (French West Indies)	141											12	10	12	9	8	11		8	7.1	7	8		9	7.6
Netherlands Antilles			15.7				13.6																		
Puerto Rico										9.2	13.4	8.8						12.2		9.3				8.8	10.2
Suriname	22.1											27											22.4		26.8
Trinidad and Tobago		16.5				18.1						27.1													15.2

Mortality

There was limited data provided within the publications covering mortality (Table 4). The ASMR values provided, however, show that within the Caribbean, the overall mortality rates were significantly high (ranging from 1.9 to 20.1 per 100,000 women), as most countries had mortality rates upward of six deaths per 100,000 women. The Bahamas reported 39 deaths for the 15-year period of 1965-1979 and experienced 23 deaths in 2018 alone. This was an exponential increase in deaths for that region. Barbados has maintained a high mortality rate over the last 15 years [14], with a rate of 9.4 per 100,000 women in 2002 and that same rate in 2018, which was greater than that of the Bahamas, which ranged from 6.2 to 7.9 per 100,000 women over that same period. Haiti had the highest death rate in the analysis, at 48.1 in 2002, which fell substantially over the same time span from 48.1 to 12.5. Over the same time period, declines were also seen, but to a considerably lesser level, in Belize, Cuba, the Dominican Republic, Guyana, and Trinidad and Tobago. From a rate of 23 per 100,000 women to 16.2 per 100,000 women, Belize dropped. The Dominican Republic decreased from 17.3 to nine per 100,000 women, whereas Cuba decreased from 8.3 to six per 100,000 women. Trinidad and Tobago decreased from 10.7 to 9.4 per 100,000 women, whereas Guyana decreased from 22.2 to 17.3 per 100,000 women. On the other arm, over the same time period, death rates significantly rose in Jamaica, Puerto Rico, and Suriname. The largest rise was seen in Jamaica, where the rate went from 12.2 to 20.1 per 100,000 women, while Suriname and Puerto Rico had significantly lower increases of 14 to 14.3 and 2.8 to 3.5 per 100,000 women, respectively.

**Table 4:** The Cervical Cancer Mortality Data (Deaths) and (Age Standardized Rate (ASMR)/per 100,000 Women) Provided by the Peer-Reviewed Publications. Bahamas [5,27,28], Barbados [5,13], Belize [13,29], Cuba [5,13,30], Dominican Republic [5,13], French Guiana [13], Grenada [16], Guadeloupe [5,13,31], Guyana [5,13], Haiti [5,13], Jamaica [5,13], Martinique [5], Puerto Rico [5,13], Suriname [5,13], Trinidad and Tobago [5,13,26]

Caribbean Territories	Cervical Cancer Deaths/Age Standardized Mortality Rate Per 100,000 Person-Year (ASMR)											
	1965	1965-1979	1995-2009		2002	2010	2016	2000-2009		2018		2020
	ASMR	Deaths	Deaths	ASMR	ASMR	ASMR	ASMR	Deaths	ASMR	Deaths	ASMR	ASMR
Bahamas		39			6.2					23	7.9	
Barbados					9.4					27	9.4	
Belize					23					25	16.2	
Cuba	20				8.3	7.1	9.1			597	6	
Dominican Republic					17.3					571	9.9	
French Guiana (French West Indies)										5	3.7	
Grenada									8.7			
Guadeloupe										19	3.3	7.9
Guyana					22.2					64	17.3	
Haiti					48.1					563	12.5	
Jamaica					12.2					361	20.1	
Martinique (French West Indies)										14	1.9	
Netherlands Antilles												
Puerto Rico					2.8					114	3.5	
Suriname					14					47	14.3	
Trinidad and Tobago			974	9.7	10.7					97	9.4	

Patient demographics

Across the 16 Caribbean territories, seven demographic factors were explored in the literature for CvC patients. These were age, marital status, ethnicity, income, highest level of education, socioeconomic status, and geographical residence (Table 5). Two-third of the articles brought in from Puerto Rico were geared toward gaining a grasp of the populace's knowledge of CvC, HPV infection, and its vaccine. This can be a useful tool for creating strategic initiatives that target particular age groups of people living in different socioeconomic strata across the Caribbean.

**Table 5:** Patient Demographics as a Disease Burden Measure Within the Peer-Reviewed Publications. x indicates the presence of the disease burden measure under consideration. Bahamas [32-34], Barbados [35], Cuba [30,36], Curacao [37,38], Dominican Republic [39], French Guiana [40-43], Guyana [44,45], Haiti [46], Jamaica [18,27,47-53], Martinique [20,54,55], Netherlands Antilles [56-63], Puerto Rico [64,65], St. Kitts and Nevis [66], St. Vincent and the Grenadines [66], Suriname [23,67], Trinidad and Tobago [68-70]

	Patient Demographics						
Caribbean Territories	Age	Marital Status	Highest Level of Education Achieved	Income	Ethnicity	Socioeconomic Status	Geographical Residence
Bahamas	x	x			x		
Barbados	x	x	x			x	
Cuba	x	x	x	x			x
Curaçao (Dutch Caribbean)	x				x		
Dominican Republic	x	x	x	x			
French Guiana (French West Indies)	x		x				
Guyana	x				x		
Haiti	x	x					
Jamaica	x	x	x	x	x		x
Martinique (French West Indies)	x	x		x		x	x
Netherlands Antilles	x	x			x	x	x
Puerto Rico	x	x	x	x			
St Kitts and Nevis	x	x	x	x		x	
St Vincent and the Grenadines	x	x	x	x		x	
Suriname	x				x		
Trinidad and Tobago	x	x			x		x

In addition to the deduction that women between the ages of 25 and 34 have the highest risk of contracting the HPV, it is believed that the target population's age has a significant impact on their level of knowledge and the actions they take in the direction of prevention and diagnosis [14]. The overall average age of diagnosis was 24-61 years, with a range from 24 to 84 in several territories.

Marital status was seen as an indicator of safer sexual practices and responsibility. Puerto Rico had the largest proportion of married people (80%) [64,65], followed by Trinidad and Tobago (72%) [68-70]. However, most studies involved half or fewer married participants. Educational attainment was also associated with knowledge, awareness, and a positive attitude towards learning. Qualitative results showed that educational attainment, awareness of the disease, and desire to take part in CvC screening or review were related.

The participants' annual income was disclosed in the publications from the Dominican Republic and Cuba [30,36,39]; however, it was only given as a range of less than or greater than $15,000/$20,000 per year; thus, it was found that socioeconomic status and income have no significant impact on whether a woman received a CvC diagnosis. However, it was expected that the patient's ability to pay for specialized and private care would have an impact on access to treatment and recovery choices such as uterine and fertility preservation.

More research on how socioeconomic status affects outcomes to determine the impact of income and socioeconomic status on both the influence of accessibility and/or limitations was found to be crucial. Healthcare, treatment, and a more active lifestyle are all more readily available to people with higher incomes. Problems with health insurance and other financial constraints are the root causes of restrictions for those in lower socioeconomic classes. Similar to this, socioeconomic position denies access and privilege to some while imposing limitations and restrictions on others. Geographical influence and socioeconomic status are closely related because a person's social standing typically determines where he lives. Depravity, luxury, and riches are all influenced by one's employment status, income, social class, and fortune [54].

Ethnicity was also shown to be associated with receiving an abnormal cervical smear, and as a result, it is thought to play a role in the development of CvC [16]. Smits et al. [71] indicated that the HPV virus mutates at an extremely slow rate, which 'coincides with the evolution of man' and is therefore considered to have evolved from the origin of man. This speculates that as man traversed the geographic landscape and propagated over time so too did the HPV virus. For the same reason, it can be deduced that the resultant diversification of man brought forth variations in HPV strains and their impact on man [67]. For example, mainly African women were impacted by CvC in Curaçao [71] and Trinidad and Tobago [37]. In a study conducted in Trinidad and Tobago during the period of 1995-2009, of 487 women, 243 were African, 125 were Indian, and 119 were of mixed descent [37]. Afro-Guyanese women are significantly more susceptible to CvC than the Indo-Guyanese and the Amerindian ethnicities in Guyana [25]. The Maroons (women) in Suriname were found to have the highest prevalence of atypical squamous or higher levels of cytological abnormalities when compared to the other ethnicities in the region. The Amerindians, although not as susceptible as the Maroons, were found to be more susceptible than the Hindustani and Javanese [16]. Jamaica further discussed ethnicity and cultural influence to be a barrier to participation in screening programs and the diagnosis of CvC [44].

Clinicopathology

The Bahamas, Curacao, the Dominican Republic, French Guiana, Guyana, Haiti, Jamaica, and Puerto Rico highlighted the need to investigate the impact co-morbidities had on the susceptibility of persons developing an HPV infection, on HPV-positive persons or persons undergoing treatment for CvC [15]. The Caribbean has been reported as a common hub for sex tourism [71], which can result in the proliferation of STDs if left unchecked. In regions such as Curaçao where it was noted to have a lower male-to-female per capita ratio as compared to other regions, the rate of illegal prostitution was expected to increase [71]. In addition, some regions utilize practices that are part of their normal personal routines, which have been noted to contribute to the development of infections that further increase the proliferation and severity of HPV infections. Haiti has reported the use of hygiene agents called twalet deba; these constitute plant and chemical-based agents such as balsam, castor oils, and Borasol. These vaginal cleansing products were attributed to the prevalence of HPV infections [17]. The comorbidities of HIV (Bahamas, French Guiana, Puerto Rico, and Haiti), AIDS (Puerto Rico) [72], and syphilis and herpes (French Guiana) were mainly discussed. The survival of persons in the Netherlands with other primary cancers was reported [29]. Of these diseases, patients with HIV as a co-morbidity are more likely to be screened using visual inspection and acetic acid application (VIA) as there is a greater probability of the expression of large lesions [73]. Four other factors identified to be precursors to initiate referrals for cytologic screening are the evidence of white vaginal discharge, the presence of dysplastic cells, postcoital and spontaneous bleeding, and discomfort during intercourse.

Behavioral risks

The age of first coitus is inversely proportionate to the probability of having a cervical neoplastic disease. The relationship is propagated by early marriages and childbearing [74]. An active sexual lifestyle from an early age was found to have a statistically significant association with HPV prevalence [38]. The number of lifetime partners was found to be proportional to HPV seropositivity and, as a result, is directly correlated to the development of HPV infection [75]. A study of 643 women in French Guiana resulted in 19.1 % of the surveyed population indicating that their age of first coitus was under 15 years. Of this population, 25.2% of the women tested positive for HPV infection, 20.5% of which were of a high-risk HPV type [40]. The use of hormonal contraceptives for more than a four-year period was noted to increase the probability of HPV-positive women developing CvC [76]. Hormonal contraception was said to act as “an enhancer of the neoplastic growth” of cervical carcinoma. The estrogen present in the contraception was stated to bind with the specific DNA that controls the transcription regulatory regions on the HPV genome [77]. This effect was said to increase with parity. The lack of awareness of HPV and its role in CvC as well as knowledge of screening and treatment options have proven to be a great challenge in the Caribbean. Although many of the publications reviewed targeted assessing the level of awareness of persons (Table 6), as well as informing them by filling any identified gaps in knowledge and clarifying misconceptions, it was still unanimously documented that there was little understanding among the public population. Smoking and alcohol use were investigated; however, no study provided data that showed a statistically significant relationship between HPV prevalence and CvC incidence.

**Table 6:** Behavioral Risks as a Disease Burden Measure Within the Peer-Reviewed Publications. x indicates the presence of the disease burden measure under consideration. Bahamas [32-34], Curacao [37,38], French Guiana [40-43],Dominican Republic [39,78], Haiti [17,79-82], Martinique [55], Netherlands Antilles [57,83], Puerto Rico [65,84-93], St. Kitts and Nevis [66], St. Vincent and the Grenadines [66], Trinidad and Tobago [5,13,14,25,26], Barbados [35,94], Cuba [30,95], Dominican Republic [96,97], Puerto Rico [98], Jamaica [48,51-53,99]

	Behavioral risks						
Caribbean Territories	Smoker	Alcohol Use	Multiparity	Age of First Coitus/Sexual Proclivity/ Multiple Sexual Partners	High-Risk Occupation	Contraceptive Use	Awareness
Bahamas	x	x	x	x		x	x
Barbados			x	x		x	x
Cuba	x			x	x	x	x
Curaçao (Dutch Caribbean)			x	x	x	x	
Dominican Republic		x	x	x	x	x	x
French Guiana (French West Indies)	x	x	x	x		x	
Haiti			x	x	x	x	x
Jamaica	x	x	x	x		x	x
Martinique (French West Indies)				x			
Netherlands Antilles	x	x		x		x	x
Puerto Rico	x	x	x	x		x	
St Kitts and Nevis			x	x		x	x
St Vincent and the Grenadines			x	x		x	
Trinidad and Tobago	x	x		x			x

Genetics

An exceptionally wide variation of HPV types exists within the Caribbean: HPV-16, 18, 33, 42, 44, 45, 51, 52, 53, 55, 56, 58, 59, 66, 68, and 70 [55]. The study of the genetic makeup of the HPV virus has led to the development of vaccines that target specific types of HPV [98,100]. This is significant as it has been identified that different countries experience varied subtypes of HPV that are most common in their region [101]. In 2006 the Bahamas noted that HPV-18 was most prevalent in its region. HPV-16 was prevalent in Curaçao, HPV-45 in Jamaica, and HPV-52 in Tobago [101]. In 2011, Suriname reported the prevalence of HPV-16, 18, and 45 [23]. French Guiana found HPV types 31, 68, and 53 most common [40]. Table 7 presents the genetics and molecular analysis in the peer-reviewed publications.

**Table 7:** Genetics as a Disease Burden Measure Within the Peer-Reviewed Publications. x indicates the presence of the disease burden measure under consideration. Bahamas [32,33], Cuba [30], French Guiana [40-43], Guyana [45], Haiti [46], Jamaica [27,47,53], Martinique [20,55], St. Kitts and Nevis [66], Trinidad and Tobago [68], Anguilla [98], Barbados [99], Belize [100], Curacao [37,38,101,102], Guadeloupe [103,104], Netherlands Antilles [57,105], Suriname [23,105], Puerto Rico [72,95,106], Suriname [107,108], Belize [109]

	Genetics and Molecular Analysis				
	Inherited		Somatic		
Caribbean Territories	HPV Positive for High-Risk HPV-16 and 18 or HPV Prevalence	Polymorphism in Codon 72 of the Tumor Suppressor Gene P53	Allelic Loss	Cellular Oncogenes	Mutations of Genes (Tumor Suppressor Genes)
Anguilla	x			x	
Bahamas	x				
Barbados	x				x
Belize	x				
Cuba	x				
Curaçao (Dutch Caribbean)	x				
French Guiana (French West Indies)	x				
Guadeloupe	x				
Guyana	x				
Haiti	x				
Jamaica	x				
Martinique (French West Indies)	x				
Netherlands Antilles	x	x	x		
Puerto Rico	x				
St Kitts and Nevis	x				
St Vincent and the Grenadines	x				
Suriname	x	x	x		
Trinidad and Tobago	x				

HPV-16 and HPV-18 account for at least 70% of all invasive CvC cases, with HPV-16 being the most predominant [45]. This confirms the range of high-risk HPV types present in the Caribbean to be HPV-16, 18, 35, 45, 51, 52, 53, and 66 [47]. In addition to the multitude of HPV types identified, it is necessary to further analyze the various mutations of the genes of the same types of HPV, such as for HPV-16 [67]. It was found that each mutation acts differently within its host, as well as its severity of impact varied with the nature of the various influencing factors identified within this review. Extra-chromosomal DNAs, such as transposons, which exist in all cell types, and small circular DNA called spcDNA are crucial in the metastasis of CvC [98]. Investigations into the gene sequencing of these molecules have allowed mapping of the phylogeny of the HPV virus, enabling a better understanding of its origin [67].

Treatment and control

CvC screening and vaccination are crucial in the control of CvC (Table 8). The HPV vaccine was licensed by the Food and Drugs Administration for girls aged nine years and older on June 8, 2006, and recommended for use from age 11-12 by the Committee on Immunization Practices (ACIP). HPV-16 and HPV-18 were identified to be the main types of HPV responsible for CvC [68]. As such, the quadrivalent HPV vaccine was recommended as the most effective as it covers the most prevalent types, as well as HPV types 6 and 11, which are low risk, but responsible for over 90% of genital warts [104]. This provides vaccine-specific cross-protection and was predicted to reduce the overall annual HPV-16/HPV-18-related CvC incidences [41]. Barbados, Belize, Guyana, Trinidad and Tobago, and the US Virgin Islands mentioned introducing this vaccine into their routine female vaccination regime, while Puerto Rico made it mandatory in order to access entry to schools [66].

**Table 8:** Control as a Disease Burden Measure Within the Peer-Reviewed Publications. x indicates the presence of the disease burden measure under consideration. Barbados [99], Belize [100,109], Curacao [37], Dominican Republic [39,77], Guyana [73,110], Haiti [46,81,111,112], Netherlands Antilles [113], Puerto Rico [64,65,84,86,89,90,114], Suriname [23,67], Trinidad and Tobago [115], US Virgin Islands [116]

	Control					
	Vaccination		Screening Method Utilized			
Caribbean Territories	Year Introduced	Age Group Targeted/Years	Visual Inspection with Acetic Acid Application, Followed by Crytotherapy	Colposcopy	Pap Smear	Biopsy/Histological Confirmation
Barbados	2013	11				
Belize	2006	9-13	x	x	x	x
Curaçao (Dutch Caribbean)					x	
Dominican Republic				x	x	
Guyana	2012	10-13	x			
Haiti			x	x	x	x
Netherlands Antilles					x	x
Puerto Rico	2018	11-12				
Suriname					x	x
Trinidad and Tobago	2013	11-13			x	
United States Virgin Islands	2006	Not Mentioned				

In tandem with the utilization of vaccination, efficiency in CvC screening leading to early detection of cytological abnormalities was identified to be imperative to furtive treatment and survival. Belize and Haiti offer all four screening methods mentioned in Table 8 to facilitate the varied needs of their population. Haiti also introduced a self-testing HPV method to provide a cost-effective approach to screening [111], while the cervical Pap smear was mentioned as available in seven territories and described as “uncomfortable, but necessary” [89].

The only regions throughout the review that spoke of CvC treatment measures were the Bahamas, Belize, Guyana, the Netherlands Antilles, and Trinidad and Tobago (Table 9). The treatment options mentioned were surgery such as the loop electrosurgical excision procedure (LEEP), irradiation therapy, and chemotherapy. The LEEP procedure and radiotherapy were noted to be the primary methods of treatment, although hysterectomy and chemotherapy were also mentioned occasionally. The Bahamas utilized a combination of external beam radiotherapy and brachytherapy irradiation techniques [113]. Information was provided on numerous patient cases and applications of irradiation as an initial course of treatment was the choice action on the first recurrent sign of cancer. Belize reported on the LEEP, radical hysterectomy, and chemotherapy as being accessible, but limited within specific regions [115]. Guyana reported mainly cryotherapy (usually carried out after VIA) and LEEP, followed by a referral for review in a year’s time. The Netherlands utilized chemo-radiation, radiotherapy, and surgery as its choices of treatment depending on the stage of cancer observed [21]. Each stage is treated with a specified combination of radiotherapy and chemotherapy or other procedures, such as lymph node dissection, as needed. The journal article from Trinidad and Tobago provided statistics on patients within the sample population who received chemotherapy, radiotherapy, and surgery. These data correlated to other variables such as geographic residence and ethnicity [73].

**Table 9:** Treatment as a Disease Burden Measure Within the Peer-Reviewed Publications. x indicates the presence of the disease burden measure under consideration. Bahamas [117], Belize [118], Guyana [110], Netherlands Antilles [56,119], Trinidad and Tobago [26]

		Treatment	
Caribbean Territories		Surgery/Leep	Irradiation
Bahamas		x	x
Belize		x	x
Guyana		x	
Netherlands Antilles		x	x
Trinidad and Tobago		x	x

## Conclusions

PubMed was found to be an excellent source of peer-reviewed publications on topics pertaining to CvC in the Caribbean. Although significant research has been conducted in the Caribbean, there still exists a substantial amount of data left to be garnered. There exist enormous gaps in clinicopathology and genetics, as well as a more detailed and specialized investigation specific to the behavioral risks and patient demographics is paramount. It was found that, even though the studies examined in the papers collected a sizable amount of data, a large portion of the data was not utilized. Thus, access to source data will allow for further valuable insights and contributions. In addition, to the measures indicated within this review, the disease burden of environmental impact was only remotely considered, as covered under behavioral risks by other studies, which identified the causative effect of smoking to be a potential cause of cervical carcinogenesis. Some countries, such as the Bahamas, Barbados, the Dominican Republic, Haiti, the Netherlands, and Trinidad and Tobago, are well on their way to mapping a sound course for the prevention and management of CvC by exploring multiple disease burden measures. However, they too require a more thorough examination of these measures, with a focus on causative agents and attitudes, not limited to HPV that must be evaluated and mitigated. Only then a pool of data from all territories can be created to equip the Caribbean region with the resources necessary to eliminate CvC.
